# Prioritising key motivators and challenges influencing informal carers’ decisions for participating in randomised trials: An embedded Study Within A before and after Trial (SWAT 55)

**DOI:** 10.12688/hrbopenres.13125.1

**Published:** 2020-10-02

**Authors:** Valerie Smith, Margarita Corry, Declan Devane, Shaun Treweek, Andrew Hunter, Susanne Grylka-Baeschlin, Kathleen Hannon

**Affiliations:** 1 School of Nursing and Midwifery, Trinity College Dublin, 24 D’Olier Street, Dublin, D02 T283, Ireland; 2 Aras Moyola, School of Nursing and Midwifery, National University of Ireland, Galway, 26 Upper Newcastle, Galway, H91 E3YV, Ireland; 3 Health Services Research Unit, University of Aberdeen, Room 306 Health Sciences Building, Aberdeen, AB25 2ZD, UK; 4 Institute of Midwifery, Zurich University of Applied Sciences, Zurich, 8401, Switzerland

**Keywords:** Study Within A Trial, informal carers, survey research, trial participation, trial design.

## Abstract

**Background:** Family members, or others, often assume the role of informal (unpaid) carers of people with chronic illnesses. Care-giving, however, can impact profoundly on the quality of life of carers and can cause carer worry, stress and guilt. Implementing interventions that positively affect the lives of carers is important; however, carers as a group are often difficult to reach. We embedded a study within a pilot-feasibility trial of a mindfulness based intervention to determine and prioritise the key motivators and challenges influencing informal carers’ decisions for participating in a trial.

**Methods:** We used a multi-method approach involving interviews with participants from a ‘ *host trial’* and data from systematic reviews to develop a survey that was distributed to informal carers in Ireland. The survey consisted of 28 motivator and 17 challenge statements. Participants rated how important they thought each statement was when deciding to take part in a trial on a 5-point Likert Scale. Mean scores and standard deviations were calculated for each statement and arranged in descending order to provide the priority lists.

**Results:** Thirty-six carers responded to the survey. Helping to create awareness about carers was the top ranked motivator, followed by four study design statements related to the time at which the study occurs, the study location, format of delivery and venue. The least important motivator related to how carers were invited to take part in a study. Difficulties in planning due to the caring role emerged as the most important challenge, followed by being unable to leave the care recipient on his/her own.

**Conclusions:** Insight into decision-making for research participation will assist trial developers tailor trial processes for informal carer populations. We recommend that trialists should consider these motivators and challenges when designing future trials involving informal carers so as to enhance trial feasibility and success.

## Introduction

The Health Research Board-Trials Methodology Research Network (HRB-TMRN) Ireland, in collaboration with the James Lind Alliance United Kingdom, participated in a priority setting partnership (PSP) to identify and prioritise unanswered questions around trial recruitment (the PRioRiTy study) ^1^. The PSP culminated in a face-to-face meeting, attended by key stakeholders (members of the public, recruiting clinicians and researchers), where a top-10 list of unanswered priority questions on trial recruitment was agreed and ranked in order of importance. Ranked highly was a question on key motivators influencing members of the public decisions for participating in randomised trials (PRioRiTy question 6) ^1^.

Family members, or others, often assume the role of informal (unpaid) carers of people with chronic illnesses. Care-giving, however, can impact profoundly on the quality of life of caregivers and can cause carer worry, stress and guilt. Family members providing unpaid care have been described *“…as a hidden patient group…”* ^2^. Mindfulness based interventions have the potential to positively impact on the lives of carers by reducing caregiver depression, anxiety and stress, and by improving carer quality of life ^3^. A randomised pilot-feasibility trial was planned by a Dublin-based university research team to test a mindfulness based stress reduction (MBSR) intervention, compared to no intervention, for informal carers of people with chronic illnesses in one region in Ireland. As informal carers represent a geographically disperse discrete group within the general public who might face specific challenges when deciding to take part in a trial, the MBSR trial presented an excellent opportunity to embed a Study Within A Trial (SWAT). The SWAT was designed to ascertain and prioritise key motivators and challenges influencing informal carers’ decisions for participating in a trial, thus helping to advance the design and conduct of future trials in this, and other, similarly discrete populations. The SWAT protocol was prospectively registered with the SWAT repository as SWAT-55.

### Context of SWAT-55

The SWAT-55 host trial was a planned pilot-feasibility randomised trial (ClinicalTrials.gov identifier NCT03048565, registered 9 ^th^ February 2017) based on the following PICO (population, intervention, comparator, outcomes);

- Population: Informal carers, defined as a person (relative, neighbour, friend or significant other) providing personal help, support or care for an individual (adult or child) with a chronic illness and who were not a paid health care provider. A person with a chronic illness was defined as an adult or child with a diagnosed condition of six months duration or longer. Access to carers was through Family Carers Ireland, a registered charity representing carers in Ireland who agreed to distribute letters of invite via email to carers in the Dublin region.
- Intervention: A MBSR programme delivered over eight weeks (two hours/week) by a trained mindfulness teacher, with participants encouraged to practice mindfulness exercises between sessions.
- Comparator: No MBSR programme.
- Outcomes: Clinical outcomes were baseline and post intervention (up to two weeks from end of programme and six months follow-up) stress, mindfulness and quality of life data collected using self-report questionnaires. Pilot measures included recruitment processes, data collection methods and intervention delivery. Feasibility outcomes were recruitment success, time to recruit, attendance at classes, dropouts and participant satisfaction.

### Host trial sample size, randomisation and recruitment

The planned sample size for the host trial, based on a recommended sample size for pilot studies of 30 per group ^4^ was 80 informal carers, or 40 per group allowing for a 25% attrition rate (10 participants per group) at six-months follow-up, randomised on a ratio of 1:1 using a computer-randomised number generator. Ethical approval to conduct the study was granted by the Research Ethics Committee of the lead researcher’s university. Recruitment to the host trial commenced in March 2017 with intervention delivery planned for April-June 2017. SWAT-55 was planned to commence in June 2017.

An invitation to participate in the host trial was emailed to 538 Dublin based registered Family Carers Ireland members. Intervention delivery was initially planned as face-to-face; however, by the end of August, despite efforts, two carers only were recruited and randomised to the intervention group, one of whom had to withdraw subsequently because he/she was unable to attend the intervention sessions. Following a Trial Steering Group (TSG) meeting, a decision was made to deliver the intervention in an online format and open the study to a wider national base. Ethical approval was granted, and an updated study invitation letter was circulated in September 2017. A social media invitation was also posted to the Family Carers Ireland Facebook page. By October 2017, 11 carers only were recruited to the study; five in the intervention group and six in the control. Following a further TSG meeting, a decision was made to change the trial design to a before and after trial, thus converting the host trial to a non-randomised pilot-feasibility trial ^5^, on the basis that a randomised trial was not feasible at recruitment level. Participants recruited to the control arm of the original trial were subsequently offered the intervention, and the revised design was further advertised. Between mid-October and end of December 2017, 17 carers were recruited to the study, 15 returned baseline data, of which seven returned end of intervention data (quality of life, mindfulness and stress outcomes). These challenges further emphasised the importance of SWAT-55 in exploring the reasons why informal carers, as a discrete group within the general population, may or may not decide to take part in a randomised trial. Although the original host trial was redesigned, we proceeded with the SWAT as planned, albeit as a study within a non-randomised trial, accepting that this deviated from our original intention of conducting the SWAT as a study within a randomised trial.

## Methods

### Design

A multi-method two-phase study was conducted. Phase 1 involved a series of face-to-face interviews with a sub-sample of participants from the host trial and a review of systematic reviews that assessed motivators or barriers for taking part in trials ^6– 12^. Resultant data from both the interviews and systematic reviews were used to collate a list of key motivators and challenges. Phase 2 was a national survey of how important these key motivators and challenges were to informal carers in making a decision on trial participation.

***Phase 1.*** The host trial consent form provided participants with an option to agree to future contact for follow-up studies arising from the trial. Of the 17 carers recruited to the host trial, 11 agreed to future contact. These 11 carers were contacted via email (addresses provided as part of the host trial) and invited to take part in an interview designed to ascertain their views of and reasons (motivators and challenges) for participating in research. We aimed to recruit 10 participants for interview; however, only two came forward. The interviews, which were semi-structured using an interview guide, were held at a mutually agreed time and venue. The interviews were conducted by the lead author, a female Prof in Midwifery with 10 years’ experience of sensitive interviewing in healthcare. The interviews were recorded and transcribed verbatim. The data was manually coded by two authors (VS and AH), and analyzed, based on a thematic analytical approach using discussion, iteration and consensus, to determine common categories of i) motivators and ii) challenges, for use in phase 2 of the study.

Seven systematic reviews of studies of reasons for participating in trials ^6– 12^ were identified in an evidence mapping exercise conducted as part of the PRioRiTy study ^1^ based on a search of MEDLINE, EMBASE, Cochrane Database of Systematic Reviews, Social Sciences Citation Index and ERIC using a combination of search terms (e.g."attitudes to trials":kw OR (participat* or recruit* or enrol* or select*) near/8 (trial* or research or study):ti) from the Cochrane systematic review of strategies to improve recruitment to trials ^13^ and from the ORRCA project ^14^. The reviews were identified as eligible for the evidence mapping exercise in the PRioRiTy study ^1 ^by at least two members of the Steering Committee. Data related to motivators/barriers for taking part in a trial were extracted onto an excel sheet for SWAT 55 by one author (VS) and corroborated by a second (AH). These data were combined with the data from the interviews to develop categories, inclusive of aligned motivator/challenge statements associated with these categories, for use in the phase 2 survey. An ‘audit trail’ of developing categories from initial codes was maintained to ensure confirmability of the process. Survey development occurred between January and June 2018.

***Phase 2.*** Phase 2 was a national survey of informal carers in Ireland to prioritise the key motivators and challenges when making decisions to participate in a trial (hypothetical or real). Developed based on the findings from phase 1, the draft survey was reviewed by a carer for user-ability and by the National Adult Literacy Agency (NALA) for plain language and comprehension. Following a number of edits and language ‘tweaks’, the survey received the Plain English Mark and was finalized. Survey distribution, using SurveyMonkey, was planned for July/August 2018; however, as Family Carers Ireland were undertaking a survey of their own at the time, distribution was delayed, initially until September 2018, and subsequently to January 2019. The original agreement was for Family Carers Ireland to distribute the survey link to their database of registered members, accompanied by the study information leaflet and the lead applicant’s contact details to discuss the study further, as needed. The survey was anonymous with no details of participant’s names, locations, email addresses, or any other identifying details requested. At this time, however, uncertainty and concerns related to the new General Data Protection Regulations (GDPR) that had come into effect in 2018 prohibited email distribution, and the survey finally went live at the beginning of February 2019 via the Family Carers Ireland Facebook page, with closure four weeks later.

### Outcome measures

The study outcomes were a prioritised list of i) motivators and ii) challenges, based on the level of importance that respondents assigned to each of the survey’s motivator and challenge statements.

### Data collection and analysis

Quantitative analytical techniques were used to aggregate individual’s ranking of motivators and challenges. Each participant was asked to rank each motivator and challenge on a 5-point Likert scale of 1=very unimportant, 2=somewhat unimportant, 3=neutral/unsure of importance, 4=somewhat important, and 5=very important. Mean scores and standard deviations (SD) for each item were calculated using SPSS (version 21). The items were subsequently arranged in descending order of importance based on the mean and SD scores attributed to them. The priority list of informal carers’ motivators and challenges for participating in trials was determined.

### Ethical statement

Ethical approval for this embedded study was granted by the Research Ethics Committee of the School of Nursing and Midwifery, Trinity College Dublin (Ref: 17/07/2017). Consent for interviews was written and taken prior to commencing the interview. Consent for the online survey was indicated by an ‘I agree’ tab, which indicated the participants gave their consent to take part in the survey.

## Results

### Interviews, systematic reviews and survey development

The interviews, involving one male and one female, were of 21 and 38 minutes duration, respectively. One participant provided full-time care, and the other part-time care. The care recipients were a child with cerebral palsy and an adult with multiple sclerosis. Although only two carers participated in the interviews, both provided valuable data for survey development. Table 1 presents examples of the categories, and associated codes, which emerged from the analysis of the interview data.

**Table 1.** Example categories and associated codes derived from interview data.

Code	Category
Interested in the topic/area that is being studied	Personal Interest/Personal Gain
Helps with reducing isolation and loneliness	
Would do me good	
How information is delivered	Trial Information
Use of simple language	
Keep it simple	
How the study information is delivered	
Use of leaflets/email	
Study location	Trial design
Prefer face-to-face	
Online is preferable	
Location where the study is being held	
Time of day that study is being held	
How the intervention is delivered	
Person/institution who is running the study	
Choice of online/face-to-face	
Topic being studied	Personal risks
Condition of care-recipient	
Unpredictability of carers role	
Getting voice heard	Carer identity
Differentiating groups (caring for child or adult)	
Feeling (under)-valued as a carer	
Might change things	Common good
Doing research is important	
Access to doctors	
Helping carers/might help carers	
Taking part in research will help carers	

The motivators and challenges data extracted from the systematic reviews, some of which overlapped with the emergent categories from the interview data, are presented in Table 2.

**Table 2.** Systematic review data.

Reference	No. of included studies	Motivators data	Challenges data
Limkakeng ^9^	14	Perceived health benefits for themselves Altruism	Mistrust of researchers Being ‘guinea pigs’ Fear of potential risks Problems with informed consent
Nalubega & Evans ^10^	21	Perceived benefits for themselves and others Previous research experience	Fear and uncertainty around taking part Disapproval by family and friends Time constraints Financial burden Lack of understanding about the research
Rivers ^7^	31	Friend/relative with previous research experience or friends/ family recommendation Accessibility - sufficient staff, services at non- traditional hours Prioritising the enrolment of minorities	Mistrust and negative perception of controlled clinical trials Lack of knowledge about ongoing research Impact of faith/religious beliefs on participation Financial constraints, lack of transportation and childcare
Wilman ^11^	49	Perceived benefits for themselves and others Being involved in decision-making Support from doctor or spouse	Research perceived as an inconvenience Concerns over risks involved Time constraints Problems with informed consent
George ^8^	44	Culturally congruent study designs Perceived benefits for themselves and others Community-based recruitment Adequate remuneration	Mistrust and consequent fear of participation Stigma related to topic of research Competing demands
Mills ^6^	33	-	Protocol issues (possibility of placebo, potential side-effects) Potential negative impact on quality of life
Tromp ^12^	38	Individual health benefits Altruism Trust in safety of research and relation to researcher Increasing comfort by participation	Fears of potential risks Distrust in research- ‘guinea pigs’ Logistics/disruption to daily life Research perceived as a burden for the participant

Using the results of the interviews and the systematic reviews, core categories, with related motivator/challenge statements, were developed. The final survey consisted of five core motivator categories with 28 associated statements and two core challenge categories with 17 associated statements. The motivator categories were; carer identity (three associated statements), study design (12 associated statements), altruism/common good (four associated statements) personal interest (six associated statements) and study information (three associated statements). The challenge categories were personal risk (11 associated statements) and study design (six associated statements).

### Survey findings

Thirty-six informal carers returned a completed survey. The majority of respondents were ≥36 years of age, female, educated to minimum leaving certificate level and unemployed ( Table 3).

**Table 3.** Respondent’s demographics.

Demographic	Category	Number (%)
Age (years)	18-25	1 (3%)
	26-35	0
	36-45	9 (25%)
	46-55	12 (33%)
	≥ 56	14 (39%)
Gender	Female	34 (94%)
	Male	2 (6%)
Education	No formal qualifications	0
	Primary or first school	0
	Group or Junior certificate, ‘O’ levels /GCSE, or equivalent	3 (9%)
	Leaving certificate, ‘A’ levels, NCVA level 1 certificate, or equivalent	11 (31%)
	Third Level Bachelor Degree	13 (37%)
	Postgraduate Master’s degree or PhD	8 (23%)
Employment status	Unemployed	14 (39%)
	Part-time paid work	7 (19%)
	Retired	4 (11%)
	Unable to work due to illness/disability	3 (8%)
	Full time paid work	3 (8%)
	Student	2 (6%)
	Casual paid work	1 (3%)
	Other (e.g. carers leave)	1 (3%)
	Not answered	1 (3%)

When asked to indicate with whom they lived, some respondents’ ticked more than one response option (e.g. lived with partner and children). Two respondents indicated living on their own, and the remainder reported living with a partner (n=22), their children (n=19), their mother (n=7), their father (n=2) and with siblings (n=2). Twenty-nine of the 36 respondents (81%) lived in the same residence as the person they were caring for. The majority of participants were more than 10 years in their caring role (61%; n=22), followed by 2–5 years (19%), 6–10 years (17%) and <2 years (3%). Thirty-three respondents (92%) reported that they provided care on a full-time basis, with the remaining three (8%) providing care part-time. The care recipients (n=38 as some respondents indicated they cared for more than one person) in most cases, were children (n=16), followed by parent(s) (n=10), partner/spouse (n=9) and sibling(s) (n=3). Five care recipients were under the age of 17 years, seven were aged 18 to 29, six were aged 30 to 39 years, and the remaining 18 were aged over 40 years. Of these 18, 14 were 60 years or older. Most respondents (78%; n=22) had not received any training for their role as an informal carer. Of those that did, some indicated previous professional training (e.g. nursing, disability or mental health) or varied short training courses, for example, manual handling, infection control, pain management or courses on autism spectrum disorder. The condition of the care recipients varied widely, with many respondents caring for people with multiple conditions, and comorbidities; for example, Alzheimer’s/Dementia/Parkinson’s (n=8), Motor Neurone Disease/Multiple Sclerosis (n=2), Autism/autistic traits (n=5), Downs Syndrome/other intellectual disability (n=6), brain/spinal injury (n=2), mental ill-health (n=2), emphysema (n=1) and cancer (n=1).

***Priority list of motivators.*** Table 4 provides the list of motivators prioritised by the mean and SD scores attributed to each motivator statement. Helping to create awareness about carers was the top ranked motivator for participating in a trial, followed by four study design categories related to a suitable time at which the study occurs, the study location, format of delivery (i.e. online) and venue. The least important motivators for deciding to participate in a trial, from the carers’ perspectives, related to study information issues; that is how they were informed of or invited to take part in the study, with all three associated statements averaging mean importance scores of 3.5 or less.

**Table 4.** Priority list of motivators.

Rank	Motivator	Mean (SD)	Category
1	The research will help create awareness about carers	4.40 (1.30)	Carer identity
2	The study is held at a time that suits me	4.39 (1.23)	Study design
3	The study is held at a place that is easy to find and easy to travel to	4.26 (1.29)	Study design
4	I can take part in the study online	4.19 (1.34)	Study design
5	The study is held at a place I feel comfortable in	4.16 (1.27)	Study design
6	Taking part will help researchers get valuable information about carers and their needs	4.15 (1.41)	Altruism/common good
7	The researchers understand the different issues carers face when caring for a younger person or an older person	4.13 (1.38)	Carer identity
8	I am very interested in the topic being studied	4.13 (1.41)	Personal interest
9	By taking part, carers might get more access to doctors or useful information	4.10 (1.32)	Altruism/common good
10	It is simple and easy to understand what is being studied and why	4.06 (1.30)	Study design
11	Doing research is important	4.06 (1.39)	Altruism/common good
12	I am interested in research on carers	4.06 (1.46)	Personal interest
13	The language used is easy to understand	4.03 (1.30)	Study design
14	The study treats carers for a younger person and carers for an older person as unique groups with different needs	4.03 (1.38)	Carer identity
15	New research might help carers in their day-to-day lives	4.00 (1.46)	Altruism/common good
16	Taking part will make my voice heard	3.97 (1.38)	Personal interest
17	I trust the institution running the study	3.97 (1.40)	Study design
18	I can choose how I take part in the study (for example, online or face-to-face)	3.90 (1.40)	Study design
19	I trust the person running the study	3.84 (1.19)	Study design
20	By taking part, I might gain access to doctors or useful information	3.80 (1.45)	Personal interest
21	Being asked to take part in the study makes me feel valued	3.74 (1.24)	Personal interest
22	Taking part in the study would benefit me socially (for example, reduce isolation or provide company)	3.55 (1.48)	Personal interest
23	I was invited to take part by a carer support group	3.48 (1.06)	Study information
24	I know the institution running the study	3.35 (1.02)	Study design
25	I can take part by talking with someone face-to-face	3.26 (0.97)	Study design
26	I found out about the study through a friend or family member	3.00 (0.97)	Study information
27	I found out about the study through a leaflet	2.97 (0.75)	Study information
28	I know the person running the study	2.84 (0.97)	Study design

***Priority list of challenges.*** Table 5 provides the list of challenges prioritised by the mean and SD scores attributed to each challenge statement. Personal risk, associated with difficulties in planning due to the caring role emerged as the most important challenge for carers when deciding on participating in a trial (mean 4.13, SD 1.25), followed by being unable to leave the care recipient on his/her own. Not knowing the person running the study was deemed to be the least important challenge for carers when deciding to take part in a trial (mean 2.74, SD 1.18).

**Table 5.** Priority list of challenges.

Rank	Challenges	Mean (SD)	Category
1	Life as a carer makes it difficult to plan ahead	4.13 (1.25)	Personal risks
2	The person I care for cannot be left alone (I do not have anyone else to take care of them)	4.09 (1.28)	Personal risks
3	Life as a carer makes it difficult to find time to take part in a research trial	4.04 (0.83)	Personal risks
4	I cannot travel to the place the study is held in	3.78 (1.28)	Study design
5	The study is held in a place I might not feel comfortable in	3.65 (1.27)	Study design
6	The language used in the study is hard to understand	3.64 (1.18)	Study design
7	I do not trust the institution running the study	3.52 (1.28)	Personal risks
8	I do not trust the person running the study	3.48 (1.28)	Personal risks
9	Taking part in a study would interfere with my daily life	3.39 (1.23)	Personal risks
10	The research does not directly affect carers	3.39 (1.31)	Personal risks
11	I do not know the institution running the study	3.09 (1.08)	Personal risks
12	I am not interested in the topic being researched	3.09 (1.35)	Study design
13	I do not believe the research will help carers	3.09 (1.51)	Personal risks
14	The topic being studied makes me uncomfortable or upset	2.96 (1.19)	Personal risks
15	I can only take part in the study online	2.96 (1.30)	Study design
16	The study requires me to talk to someone face-to-face	2.95 (1.13)	Study design
17	I do not know the person running the study	2.74 (1.18)	Personal risks

## Discussion

This embedded study within a non-randomised pilot feasibility trial has identified and highlighted important factors from the perspectives of carers that influence decision-making on trial participation. As the focus of healthcare internationally is to increase community-based care and avoid admission to secondary healthcare facilities for as long as possible ^15^, compounded by a rapidly increasing older person population ^16^, many individuals may find themselves in a caring role for which they are ill-prepared ^17, 18^. Evaluating interventions to support informal carers psychologically, socially, physically, or otherwise, is important. As a discrete group within the general population informal carers can be a hard to reach population, not least of all because of regional dispersity ^19^ and the cost, time and inconvenience that might be associated with taking part in a trial ^19, 20^. Furthermore, where trials do recruit, attrition rates in studies on carers can be high, for example, from 25% to >40% across studies ^21, 22^.

Understanding informal carers’ views of research will assist trial developers, and other researchers, accommodate their unique needs. Carer identity, specifically, research that will help raise awareness about carers was the number one collectively identified motivator for trial participation. This supports the earlier quote describing informal carers as a ‘ *hidden patient group’* ^2^ and gives consideration to a sense of isolation or loneliness that carers may experience in their caregiving roles. Trial developers and researchers, in efforts to enhance participation in trials involving informal carers, need to consider the explicit demands that the caring role places on carers. These, in particular, identified in this study, were difficulties with planning ahead (number 1 priority challenge) and being unable to leave the care recipient alone, or for any length of time, all of which have implications for the design of any future, similar, host trial.

### Implications for a future host trial

The non-feasibility of the host trial in recruiting sufficient participants implies that any plan for a future, similar trial needs to take cognisant of carers’ motivators and challenges, and would require a major rethink as to how the trial would be designed and implemented. For example, how informal carers come to know of a study (e.g. through a family member or friend, or through a leaflet), or their awareness of those conducting the study, were ranked overall, as being neutral or unimportant when deciding to take part in a trial. Thus advertising/invitational methods will not necessarily optimise recruitment to a future trial, rather other aspects of trial design, such as the value the trial has for carers’ identity, and how the intervention is delivered will play a greater role. Carers in this study have clearly identified that their caring role leaves it difficult for them to plan ahead, to have time away from their care recipient, or to travel to study locations. This implies that a trialist needs to consider either offering the intervention online or, if offering it face-to-face, either i) cover the costs for alternative care for the care recipient, and time and travel costs or ii) have the interventionist travel to the participants homes or other location convenient to them. Given that the online format only marginally increased recruitment to the host trial, the latter options would appear preferable. These will have resource implications, however, in terms of personnel required and expense, which should be factored in to the future trial’s processes and budget. Flexibility in delivering the intervention is also required, and may involve late cancellations and rearranging interventionist visits. This will have implications on the time taken to deliver the intervention, and thus trial duration, an important issue to consider in future trial plans.

### Challenges to conducting the original planned SWAT 55

A number of challenges in conducting the originally planned SWAT were encountered which may limit the results. These included limited recruitment to the host randomised trial and a redesign to a before and after trial resulting in a reduced purposive sampling frame for phase 1 of the planned SWAT. Although rich data were provided by the two interviewees from the before and after trial, a wider pool of participants might have better ensured, or at least increased our confidence in data sufficiency. This was overcome somewhat, however, by the extraction of data from related systematic reviews, combining these data with the interview data in developing the survey.

Despite extensive efforts, 36 participants only responded to the national survey, which is likely to represent a very small proportion of the population of informal carers across Ireland. Although we cannot be sure, the inability of Family Carers Ireland to distribute the survey via their email list, and the move towards distribution via Facebook (with only 12 ‘shares’ and 14 ‘likes’ noted), may have impacted on the survey response rate. We need to consider that had a larger number responded, providing greater caregiver representation, the ranked priority list of motivators and challenges might ultimately be different. The difficulties encountered with recruitment, however, could relate to how individuals identify with the concept of caring as evidence suggests that many individuals do not formally identify themselves as a carer or in a caregiving role ^23^. For example, in a qualitative study of 40 relatives or friends, the researchers concluded that “…self-identification with the role and label of carer is nuanced, shifting and variable” ^24^ and refers to other studies that have shown variation in how relatives identify with the term carer ^25, 26^. In one study, for example, exploring recruitment with carers of people with multiple sclerosis, participants suggested using the term ‘support’ or ‘assistance’ in place of the term ‘caregiver’ and highlighted that many people would not consider themselves to be carers ^23^. This is an important consideration for researchers when advertising trials to an already hard to reach population, and how carers, including their associated roles, are described may need to be considered within trial participant information.

## Conclusion

Insight as to the motivators and challenges that influence informal caregivers’ decisions for research participation, as offered by this embedded study, will assist trial developers and researchers to better tailor trial design and associated processes for informal caregiver populations, and adds to what is a limited evidence base. Consideration of these motivators and challenges to enhance participation has the potential to increase trial feasibility and success and reduce research waste. We recommend that trial developers consider these motivators and challenges when designing future trials involving informal caregivers.

## Data availability

### Underlying data

Access to interview transcripts are restricted under Research Ethics Committee approval as even de-identified transcript data may contain information that could potentially identify a participant based on statements made, phrasing, or personal data contributions which presents a potential breech in GDPR assurances. Transcripts may be made available, in full or in part, on individual request and only with the explicit permission of an interviewee based on the acceptability of the nature and reason for the request. Such requests such be made to the corresponding author ( smithv1@tcd.ie) in the first instance.

Zenodo: SWAT 55 survey and dataset. https://doi.org/10.5281/zenodo.4029385 ^27^.

This project contains the following underlying data:

- SWAT 55 Survey (participant responses).xlsx

### Extended data

Zenodo: SWAT 55 survey and dataset. https://doi.org/10.5281/zenodo.4029385 ^27^.

This project contains the following extended data:

- Interview Schedule.docx

SWAT Survey (Final Version).pdf

Data are available under the terms of the Creative Commons Attribution 4.0 International license (CC-BY 4.0).
