# Supplementary material for: Prioritising key motivators and challenges influencing informal carers’ decisions for participating in randomised trials: An embedded Study Within A before and after Trial (SWAT 55)
Source: HRB Open Res. 2021 May 28;3:71. Originally published 2020 Oct 2. [Version 2] doi: 10.12688/hrbopenres.13125.2 (PMC11196933; doi:10.12688/hrbopenres.13125.2)
Supplement: Peer review history

# Reviewer report: Reviewer response for version 2

**DOI:** 10.21956/hrbopenres.14395.r40134

**Published:** 2024-06-27

**Authors:** Camilla Malm

**Affiliations:** 1 Linnaeus University, Växjö, Sweden

Dear authors,

 It has been a pleasure to read your article about this important topic. The article is well-written and makes an important contribution to the under-researched field of informal carer involvement in research/trials. The study has some weaknesses, mainly the low number of respondents in the different steps of the data collection. This is partly acknowledged, discussed, and justified, which increases the trustworthiness of the study, but transparency needs to be increased.

  

1. The literature provided is relevant and fits the content of the article. However, since it has been some years since it was originally written and reviewed, I suggest that the authors perform a new literature search to see if any new articles within the topic of informal carer involvement in trials have been published.

Please take a look at my article, even though it is aimed at research more generally and not specifically towards trials it might be of some interest: [1]

  

1. The study design is partly appropriate and technically sound. The authors acknowledge the sample size in Phase 1 being too small, hence deciding to also draw on data from systematic reviews. This is acceptable and also shows the difficulties of recruiting informal carers. The authors discuss and justify their decision in a transparent way. However, when scrutinizing the systematic reviews chosen, it becomes clear that these do not focus on informal carers. Since motivators/barriers may not be the same for informal carers as for other population groups (e.g., patients), this may be a potential flaw. For example, by including literature reviews regarding informal carers, motivators regarding the person cared for would most certainly emerge. At least, the lack of systematic reviews focusing on informal carers needs to be mentioned and discussed. Regarding Phase 2, the draft survey being reviewed by only one carer for usability is a bit meager and needs to be justified.

 

1. Sufficient details of methods and analysis are provided to allow replication by others. Perhaps it should be more clearly mentioned that the quantitative analysis is limited to descriptive statistics. Also, the “Data collection and analysis” section should include the qualitative part of the study (interviews + thematic analysis). In the present version, this is mentioned under Phase 1. It would be feasible to add relevant methodological references to this section.

 

1. The statistical analysis and its interpretation are, to the best of my knowledge, appropriate, even though the small number of respondents limits the generalizability of results.

 

1. The quantitative source data underlying the results are available to ensure full reproducibility. Transcripts from the interviews may be made available on request.

 

1. The conclusions drawn are adequately supported by the results. The authors acknowledge that the limited data limits the generalizability of results but cautiously draw a conclusion about motivators and challenges for participation at the same time as they emphasize that further research is required. I particularly like the discussion about defining oneself as an informal carer; this is really important when it comes to recruitment.

Is the work clearly and accurately presented and does it cite the current literature?

Partly

If applicable, is the statistical analysis and its interpretation appropriate?

Yes

Are all the source data underlying the results available to ensure full reproducibility?

Yes

Is the study design appropriate and is the work technically sound?

Partly

Are the conclusions drawn adequately supported by the results?

Yes

Are sufficient details of methods and analysis provided to allow replication by others?

Partly

Reviewer Expertise:

Informal caring, informal carer involvement in research and R&D

I confirm that I have read this submission and believe that I have an appropriate level of expertise to confirm that it is of an acceptable scientific standard, however I have significant reservations, as outlined above.

## References

[1] : What motivates informal carers to be actively involved in research, and what obstacles to involvement do they perceive?. *Res Involv Engagem* .2021;7(1) : 10.1186/s40900-021-00321-x 80 10.1186/s40900-021-00321-x 34749834 PMC8574014

# Reviewer report: Reviewer response for version 2

**DOI:** 10.21956/hrbopenres.14395.r40138

**Published:** 2024-06-24

**Authors:** Val Morrison

**Affiliations:** 1 Bangor University, Bangor, Wales, UK

Influences on trial participation amongst informal carers/caregivers  is an important topic given the challenges  seen in study recruitment, and growing interest in carers research and intervention. Whilst well written and presented there are some flaws that limit the generalisability of findings  as presented in the discussion and study conclusions.

**In phase 1**, a multi method approach is taken. This  draws firstly from an *N of only 2* individuals recruited from those originally in the mindfulness trial (already a biased sample as they have taken part in a trial, this needs noted), with their data subjected to 'thematic analysis' - the relatively limited data would not achieve *saturation* and there is a lack of detail around the *process taken during thematic analysis*- was Braun & Clarke framework followed?  Secondly reviews of trial recruitment barriers and facilitators are analysed without noting that the *reviews are not specific to caregiving sample*s. Some acknowledgement of this is needed. In addition there are likely *condition-specific factors* that may influence perceived barriers/motivators and also some trial specific features e.g. behavioural trial, drug trial. 

 I was surprised that the issue of 'carer' labelling did not come up in the interviews ie 'I do not take part as I do not consider myself a 'carer' for my spouse/child etc . This point is raised in the discussion, but it is worth noting also that participants in Phase 1 interviews and Phase 2 surveys have already identified as carers as they are part of 'Family Carers Ireland' and so there may be a bias in this already

**The phase 2 survey** has an acknowledged very disappointing response rate and the study thus *lacks power*. I also wondered if authors could *contextualise and critically compare the carer survey findings with those of the systematic reviews of non-carer samples* analysed in Phase 1 - given that the survey was designed following review of non-carer data (systematic reviews) and only 2 carer interviews is it possible that some carer-specific motivators and challenges were not included? e.g. 'carer' label issue. More could be made perhaps of what  motivators and challenges are 'unique' to carers when recruitment to trials is sought i.e. that differ from recruiting noncarers to trials?  This would add value.

 The limited sample also makes any *subgroup analysis* e.g. by care recipient  condition, by carer gender, or by type of trial online/not etc, impossible.

 I feel these limitations need to be acknowledged and used to further  temper the conclusions if this paper is published.

Is the work clearly and accurately presented and does it cite the current literature?

Yes

If applicable, is the statistical analysis and its interpretation appropriate?

No

Are all the source data underlying the results available to ensure full reproducibility?

Yes

Is the study design appropriate and is the work technically sound?

Partly

Are the conclusions drawn adequately supported by the results?

Partly

Are sufficient details of methods and analysis provided to allow replication by others?

Partly

Reviewer Expertise:

Health psychology, caregiving in the context of physical health conditions

I confirm that I have read this submission and believe that I have an appropriate level of expertise to confirm that it is of an acceptable scientific standard, however I have significant reservations, as outlined above.

# Reviewer report: Reviewer response for version 2

**DOI:** 10.21956/hrbopenres.14395.r29557

**Published:** 2021-06-17

**Authors:** Elizabeth Randell

**Affiliations:** 1 Centre for Trials Research, Cardiff University, Cardiff, UK

Many thanks to the authors for their responses.

Is the work clearly and accurately presented and does it cite the current literature?

Yes

If applicable, is the statistical analysis and its interpretation appropriate?

Yes

Are all the source data underlying the results available to ensure full reproducibility?

Partly

Is the study design appropriate and is the work technically sound?

Yes

Are the conclusions drawn adequately supported by the results?

Partly

Are sufficient details of methods and analysis provided to allow replication by others?

Partly

Reviewer Expertise:

Intellectual disability and autism

I confirm that I have read this submission and believe that I have an appropriate level of expertise to confirm that it is of an acceptable scientific standard.

# Reviewer report: Reviewer response for version 1

**DOI:** 10.21956/hrbopenres.14235.r28738

**Published:** 2021-01-28

**Authors:** Terry A Badger

**Affiliations:** 1 College of Nursing, University of Arizona, Tucson, AZ, USA

A clearly presented SWAT, including providing insight why carers do not participate in clinical trials. There are a number of limitations to this study, especially the phase 1. Findings from two carers are unlikely to have achieved data saturation calling into question using this information to develop the survey. It would be important to have more detail about the interview methods and how authors maintained rigor.

 Recruitment from a national network was good, however the fact that so few responded in of itself is problematic.

 Surveys are usually not well received, it would be helpful to know what other methods were used beyond mailing them. The discussion is too broad and goes beyond the data. Likely this study needs to be replicated with a larger sample in both phase 1 and two.

 Manuscript would benefit from revision.

Is the work clearly and accurately presented and does it cite the current literature?

Yes

If applicable, is the statistical analysis and its interpretation appropriate?

Partly

Are all the source data underlying the results available to ensure full reproducibility?

Partly

Is the study design appropriate and is the work technically sound?

Yes

Are the conclusions drawn adequately supported by the results?

Partly

Are sufficient details of methods and analysis provided to allow replication by others?

Partly

Reviewer Expertise:

PsychoOncology: Symptom management interventions for Cancer Survivors and Caregivers.

I confirm that I have read this submission and believe that I have an appropriate level of expertise to confirm that it is of an acceptable scientific standard, however I have significant reservations, as outlined above.

# Response

**Published:** 2021-02-07

**Authors:** Valerie Smith

**Affiliations:** Trinity College Dublin, Ireland

Thank you very much for your review and valuable comments on our manuscript. We have addressed these and provide point by point responses as below.

 A clearly presented SWAT, including providing insight why carers do not participate in clinical trials

**Response**: Thank you

 There are a number of limitations to this study, especially in phase 1. Findings from two carers are unlikely to have achieved data saturation calling into question using this information to develop a survey. It would be important to have more detail about the interview methods and how authors-maintained rigor.

**Response**: Yes, our interviews were unlikely to achieve data saturation or even sufficiency, which is why we additionally drew on the evidence from systematic reviews; we have emphasised this further by adding a sentence that we made a necessary pragmatic decision to use data from systematic reviews to inform the development of the survey in phase 2 due to the limited participation in the interviews (Design section, third paragraph, lines 1-2)

 Recruitment from a national network was good, however, the fact that so few responded itself is problematic. Surveys are usually not well received; it would be helpful to know what other methods were used beyond mailing them.

**Response**: Other than a request to snowball, we didn’t go beyond the Family Carers Ireland network in advertising our survey. We recognise, in hindsight, that this may have helped. We have added a sentence to the limitations section to this effect.

 The discussion is too broad and goes beyond the data.

**Response**: The Discussion (in particular the implications section) and the Conclusion (including Abstract) sections has been amended (as per tracked changes) to address this point.

 Likely this study needs to be replicated with a larger sample in both phase 1 and two.

**Response**: Thank you. We have noted this as a recommendation now (Conclusion section). We will plan for this opportunity as it might arise in a future trial.  

 Manuscript would benefit from revision.

**Response**: We have made revisions to the manuscript as per responses above.

# Reviewer report: Reviewer response for version 1

**DOI:** 10.21956/hrbopenres.14235.r28455

**Published:** 2020-12-14

**Authors:** Elizabeth Randell

**Affiliations:** 1 Centre for Trials Research, Cardiff University, Cardiff, UK

A clearly presented SWAT building on available data to provide insight into participation of informal care givers. This is a really interesting area on which to conduct this piece of research. The authors have identified the limitations in the study design but I would add that the sample from phase 1 was very small. I would be wary of taking the findings purely from these 2 interviews forward to form the basis of the survey.

 I think the manuscript could describe the content of the interview schedule briefly to inform the reader as to how this was devised.

 Recruitment was from the Family Carers Ireland network - again, the authors have discussed this as a limited source of recruitment and perhaps this was a group of people already motivated the population in terms of making their voices heard. Did the authors consider how other carers might be reached? Collection and reporting of ethnicity data could also have been included as we consider how to reach under represented populations.

 Further description could have been included with regards to any differences in motivators or challenges depending on the type of trial carers might take part in i.e. CTIMP versus non-CTIMP? The manuscript would benefit from having survey questions included.

 I think that the results have been generalised to all carers too much and that there is important further research to be undertaken to examine how barriers faced might vary according to the needs of the individual being cared for e.g. issues around capacity and consent.

Is the work clearly and accurately presented and does it cite the current literature?

Yes

If applicable, is the statistical analysis and its interpretation appropriate?

Yes

Are all the source data underlying the results available to ensure full reproducibility?

Partly

Is the study design appropriate and is the work technically sound?

Yes

Are the conclusions drawn adequately supported by the results?

Partly

Are sufficient details of methods and analysis provided to allow replication by others?

Partly

Reviewer Expertise:

Intellectual disability and autism

I confirm that I have read this submission and believe that I have an appropriate level of expertise to confirm that it is of an acceptable scientific standard, however I have significant reservations, as outlined above.

# Response

**Published:** 2021-02-07

**Authors:** Valerie Smith

**Affiliations:** Trinity College Dublin, Ireland

Thank you very much for your review and valuable comments on our manuscript. We have addressed these and provide point by point responses as below.

 A clearly presented SWAT building on available data to provide insight into participation of informal care givers. This is a really interesting area on which to conduct this piece of research.

**Response**: Thank you

 The authors have identified the limitations in the study design, but I would add that the sample from phase 1 was very small. I would be wary of taking the findings purely from these 2 interviews forward to form the basis of the survey.

**Response**: Thank you; we have added a sentence to emphasise that we made a necessary pragmatic decision to use data from systematic reviews to inform the development of the survey in phase 2 (Design section, third paragraph, lines 1-2)

 I think the manuscript could describe the content of the interview schedule briefly to inform the reader as to how this was devised.

**Response**: The interview schedule is available as an extended File; we have now inserted the link to this file in the text under the Phase 1 subsection of the Design section (available at: http://www.doi.org/10.5281/zenodo.4029385)

 Recruitment was from the Family Carers Ireland network - again, the authors have discussed this as a limited source of recruitment and perhaps this was a group of people already motivated the population in terms of making their voices heard. Did the authors consider how other carers might be reached?

**Response**: Thank you, this is a really important point; we didn’t go beyond Family Carers Ireland other than to request snowballing. We have added an additional sentence to the limitations section to address and clarify this.

 Collection and reporting of ethnicity data could also have been included as we consider how to reach under-represented populations.

**Response**: Thank you; we did not collect ethnicity data, but certainly will consider this in any future SWAT-55.

 Further description could have been included with regards to any differences in motivators or challenges depending on the type of trial carers might take part in i.e. CTIMP versus non-CTIMP? The manuscript would benefit from having survey questions included.

**Response**: We have added this important point as a recommendation for future research (as point below). The full survey document is available as an Extended File (with the access link provided in this section; http://www.doi.org/10.5281/zenodo.4029385)

 I think that the results have been generalised to all carers too much and that there is important further research to be undertaken to examine how barriers faced might vary according to the needs of the individual being cared for e.g. issues around capacity and consent.

**Response**: We have edited the Discussion (in particular the implications section) and the Conclusion sections (including in the Abstract) to address this point. We have also included the important point about the need for further research which would examine how barriers faced might vary according to the needs of the individual being cared for (Conclusion, last line).
